# Narrowing it down: deciphering a narrow leaf phenotype in wheat

**DOI:** 10.1093/plphys/kiag103

**Published:** 2026-02-27

**Authors:** Rose McNelly

**Affiliations:** Assistant Features Editor, Plant Physiology, American Society of Plant Biologists, Rockville, United States; John Innes Centre, Norwich Research Park, Norwich NR4 7UH, United Kingdom

Wheat is a globally important crop, contributing around 20% of our dietary calories (Shiferaw et al 2013). As the population grows, total wheat production needs to increase by 43% (Hatfield and Beres 2019). One strategy to increase yield is to alter plant architecture. Donald (1968) suggested that narrow, erect leaves would be part of an ideal wheat architecture as it would optimize light capture and photosynthetic rate. Indeed, experiments comparing yields of erect-leafed versus flat-leafed wheat show a 24% increase in yield for erect-leafed plants (Richards et al 2019).

Few genes influencing wheat leaf architecture have been characterized. Liu et al (2019) identified *SQUAMOSA PROMOTER BINDING-LIKE 8*, which when knocked out alters auxin and brassinosteroid signaling, resulting in erect leaves due to a shortened lamina joint. Meanwhile, Du et al (2024) describe a narrow-leafed mutant with an amino acid substitution in WALL ASSOCIATED KINASE 2 (WAK2). The amino acid substitution facilitates WAK2 degradation through the proteasomal pathway, resulting in lower WAK2 abundance. A downstream consequence of less WAK2 is upregulation of genes involved in cytokinin degradation; for instance, *CYTOKININ DEHYDROGENASE 11* (*CKX11*) was upregulated by 80% and *CKX9* was upregulated by 300%. Consistent with this, cytokinin levels are reduced in *wak2* plants. The reduction in cytokinin influences the narrow-leaf phenotype, as application of synthetic cytokinin to *wak2* plants partially restores the phenotype. Besides these, many quantitative trait loci influencing leaf traits have been uncovered (Isidro et al 2012; Chen et al 2022), but the causative genes are unknown. Identifying additional genes that influence leaf traits will improve our ability to breed for improved leaf architecture in wheat.

In a recent study published in *Plant Physiology*, Li et al (2026) identify a wheat mutant with narrow leaves, which they name *nl1*. Compared with the wild type, the *nl1* mutant has narrower leaves from week 9 of growth. The difference became more pronounced over developmental time, and at maturity *nl1* flag leaves are 60% narrower than the wild type. Unfortunately, the *nl1* plants had significantly reduced spikelet number, grain number per spike, and thousand-grain weight, suggesting these *nl1* plants may not be beneficial in agronomic settings. As the *nl1* line arose from an EMS mutagenesis population, the plants are likely to have many additional mutations. It would be interesting to eliminate background mutations that do not cause the narrow-leaf phenotype and discover if this improves the agronomic performance of *nl1* plants.

The authors performed microscopic analysis to try to uncover the reason behind the narrow leaves in *nl1* plants. The number of epidermal cells in the upper epidermis of *nl1* flag leaves was 27.6% less than in the wild type, suggesting that *nl1* plants have defects in cell division. Although cell division was affected, there were no apparent defects in cell expansion, as cell size in *nl1* stems did not significantly differ from the wild type. Interestingly, *nl1* plants had a 40% reduction in the small vein number. Hence, the narrow-leaf phenotype may be a consequence of the reduced number of small veins, yet the molecular mechanism underlying this has not been investigated.

To identify the causative gene of the narrow-leaf phenotype, map-based cloning was conducted (Fig. 1). Using a biparental mapping population and single nucleotide polymorphism (SNP) genotyping, the authors concluded that the causative mutation was on chromosome 1B. Bulk segregant analysis, where pools of plants with either the narrow-leaf or normal-leaf phenotype were subjected to exome capture and sequencing, identified 3 closely linked SNPs. Only 1 SNP co-segregated with the narrow leaf phenotype, so this was likely to be the causative mutation.

**Figure 1:**
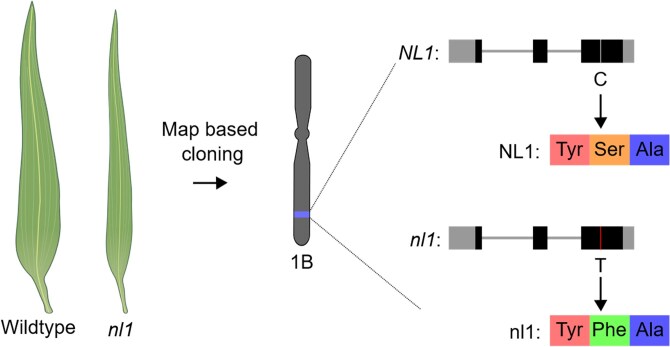
Identification of the causative mutation underlying *nl1*. The *nl1* mutant has narrow leaves. Map-based cloning identified a region on chromosome 1B that was associated with the phenotype. The phenotype segregated with a cytosine to thymine mutation, which resulted in a serine-to-phenylalanine substitution in the nl1 protein. Image created using Inkscape.

The causative SNP was within a gene (*NL1*) that encodes for a CELL DIVISION CYCLE 48-like (CDC48-like) protein. The SNP resulted in a serine-to-phenylalanine amino acid substitution in the encoded protein (Fig. 1). To confirm this mutation was causing the narrow-leaf phenotype, *nl1* plants were transformed to express wild-type *NL1* under the native and ubiquitin promoters. All these plants had normal-width leaves, confirming the point mutation in the B genome copy of *NL1* is causing the narrow-leaf phenotype. It is surprising that a single point mutation in 1 homeolog of *NL1* causes such a strong phenotype, considering that there are 2 highly similar homeologs present in wheat.

Transcriptome analysis was conducted to investigate changes in gene expression in *nl1* plants. There were 3801 differentially expressed genes between *nl1* and wild-type plants, some of which were further validated using reverse transcription quantitative PCR. Notably, there was upregulation of genes associated with cell division; one of these was cyclin E, which promotes entry into S phase of the cell cycle (Shimotohno et al 2021). The authors postulate that upregulation of cell division components may lead to defects in the assembly of cell division protein complexes, which would inhibit division; however, this hypothesis has not been experimentally tested. Furthermore, several of the differentially expressed genes were associated with cytokinin metabolism. There was upregulation of the cytokinin oxidases *CKX4* and *CKX5*, which may explain the decrease in the amount of the cytokinins trans-zeatin (tZ) and trans-zeatin riboside (tZR) in *nl1* plants. Surprisingly, the amount of cZ-riboside was increased in *nl1* plants. The biological reasoning for this is unclear, but cZ-riboside is less active (Kieber and Schaller 2018) so it is likely that total cytokinin activity is reduced in *nl1* plants. When combined with the results of Du et al (2024), this strongly suggests that reduced cytokinin levels can cause narrow leaves, so future efforts to generate plants with narrow leaves could focus on manipulating cytokinin metabolism.

Next, the authors explored existing wheat genomes and pangenomes for *NL1* variation. Four distinct *NL1* haplotypes were identified, which the authors name 1 to 4. To test the effect of the *NL1* haplotypes, 109 accessions containing haplotypes 1 to 3 were grown in the field. The authors highlight haplotype 2 as a favorable as these plants had narrower flag leaves, and some had increased tiller number. Comparison of haplotype frequencies shows that haplotype 2 has been selected for during domestication; it occurs at frequencies of 12% in populations of hexaploid wheat but 47% in modern hexaploid cultivars. The frequency of haplotype 2 could be further increased in modern cultivars by designing markers against the haplotype 2 variation identified here and incorporating these into breeding programs. Moreover, comparing the global distribution of *NL1* haplotypes could reveal which regions lack or have lower frequencies of haplotype 2 and hence where breeding for haplotype 2 could be most beneficial.

In summary, Li et al (2026) identify a point mutation in *NL1* in wheat that causes a narrow-leaf phenotype by affecting cell division and plant hormone pathways. At a molecular level there is still much to learn. For example, how does this amino acid substitution impact cell division? If equivalent mutations were introduced in the A and D homeologs, would the narrow-leaf phenotype be even stronger? Or would there be too many pleiotropic effects, or would this be lethal? Answering these questions may allow us to understand how to engineer wheat with a more ideal leaf architecture.

## Data Availability

No new data were generated or analyzed in support of this research.
